# Implications of AMPK in the Formation of Epithelial Tight Junctions

**DOI:** 10.3390/ijms19072040

**Published:** 2018-07-13

**Authors:** Pascal Rowart, Jingshing Wu, Michael J. Caplan, François Jouret

**Affiliations:** 1 Groupe Interdisciplinaire de Génoprotéomique Appliquée (GIGA), Cardiovascular Sciences, University of Liège (ULiège), Avenue de L'Hôpital 11, 4000 Liège, Belgium; pascal.rowart@uliege.be; 2 Department of Cellular and Molecular Physiology, Yale School of Medicine, New Haven, CT 06520, USA; jingshing.wu@yale.edu (J.W.); michael.caplan@yale.edu (M.J.C.); 3 Division of Nephrology, Centre Hospitalier Universitaire de Liège (CHU of Liège), University of Liège (CHU ULiège), 13-B4000 Liège, Belgium

**Keywords:** AMPK, tight junctions, epithelial cells, ZO-1, par complex, MDCK, nectin-afadin, adherent junctions

## Abstract

Tight junctions (TJ) play an essential role in the epithelial barrier. By definition, TJ are located at the demarcation between the apical and baso-lateral domains of the plasma membrane in epithelial cells. TJ fulfill two major roles: (i) TJ prevent the mixing of membrane components; and (ii) TJ regulate the selective paracellular permeability. Disruption of TJ is regarded as one of the earliest hallmarks of epithelial injury, leading to the loss of cell polarity and tissue disorganization. Many factors have been identified as modulators of TJ assembly/disassembly. More specifically, in addition to its role as an energy sensor, adenosine monophosphate-activated protein kinase (AMPK) participates in TJ regulation. AMPK is a ubiquitous serine/threonine kinase composed of a catalytic α-subunit complexed with regulatory β-and γ-subunits. AMPK activation promotes the early stages of epithelial TJ assembly. AMPK phosphorylates the adherens junction protein afadin and regulates its interaction with the TJ-associated protein zonula occludens (ZO)-1, thereby facilitating ZO-1 distribution to the plasma membrane. In the present review, we detail the signaling pathways up-and down-stream of AMPK activation at the time of Ca^2+^-induced TJ assembly.

The correct establishment and maintenance of cell-cell contacts and cell polarity in multicellular organisms are crucial for normal cell physiology and tissue homeostasis [1,2]. Epithelial cells form barriers that protect and separate multicellular organisms from the external environment. Such compartmentalization provides different internal and external environments with specialized functions [3,4]. In addition to its compartmentalization role, epithelial cell membrane integrity plays a major role in the defense against pathological organisms, as well as against disease development. Hence, the loss of cell polarity and membrane disruption are observed in cancer, acute kidney injury, apoptosis, and infection, as well as at the initial stages of some central nervous system neoplasia [5,6,7,8,9].

To maintain these two distinct regions and to protect the organism, epithelial cells are sealed together by a junctional complex formed by four main components located along the apico-basal axis, as follows: (1) TJ; (2) adherens junctions (AJ); (3) gap junctions; and (4) desmosomes. Disturbances in the formation and maintenance of TJ are observed in many pathological conditions, including cancer [10,11]. For this reason, a better understanding of their regulation may lead to novel targeted therapies [12]. Many factors have been identified as modulators of the assembly and disassembly of TJ. AMP-Activated protein kinase (AMPK) has emerged as one of these. Indeed, epithelial cells are involved in electrolyte and fluid transport across the apical and basal membrane, which consume a major part of an epithelial cell’s internal energy currency, namely its stores of Adenosine triphosphate (ATP) [13]. AMPK is the main energy sensor in all eukaryotic cells that regulate their levels of ATP. During cellular stresses such as hypoxia, starvation, glucose deprivation, or muscle contraction, the ratio of ADP/ATP or AMP/ATP will change. To restore the cellular energy balance, AMPK will promote catabolic pathways and inhibit anabolic pathways [14]. Interestingly enough, the depletion of ATP results in the rapid dislocation of cellular tight junctions (TJ), whereas ATP repletion induces a recovery of tight-junction integrity [15]. As an example, in case of kidney ischemia, TJ disassembly between proximal tubule cells allows the paracellular backleak of the ultrafiltrate into the interstitium, which in turn aggravates the renal hypoperfusion [16]. Zhang et al. were the first to demonstrate a role of AMPK in the regulation of epithelial tight junction assembly and disassembly. Note that the role of AMPK in TJ regulation appears to be independent of intracellular [ATP] levels. These observations opened new investigations into the mechanisms through which AMPK serves at the crossroads between the regulation of cellular energy and TJ homeostasis [17].

## 1. TJ Are Multiprotein Complexes

The concept of TJ (also known as *zonula occludens*) emerged in 1963 with Farquhar and Palade’s experiments demonstrating, by electron microscopy, the regular occurrence in various rat and pig epithelia of a characteristic junctional complex whose components bear a relationship to each other and to the lumen of the organ [18]. Briefly, an individual TJ strand is associated laterally with another TJ strand on adjacent cells to form paired strands, where the extracellular space at this region is completely obliterated [19]. TJ are the most apical constituent of the junctional complex with AJ immediately underneath [20]. TJ can be thought of as gaskets that define and seal the most apical border of cell-cell contacts. For several years following their morphological observation, TJ were further investigated and major progress was achieved by Stevenson et al. in 1986. They explored the biochemical structure of TJ and identified ZO1 as one of their protein components [21], which has gone on to serve as a canonical marker for the assessment of TJ formation.

**Figure 1:**
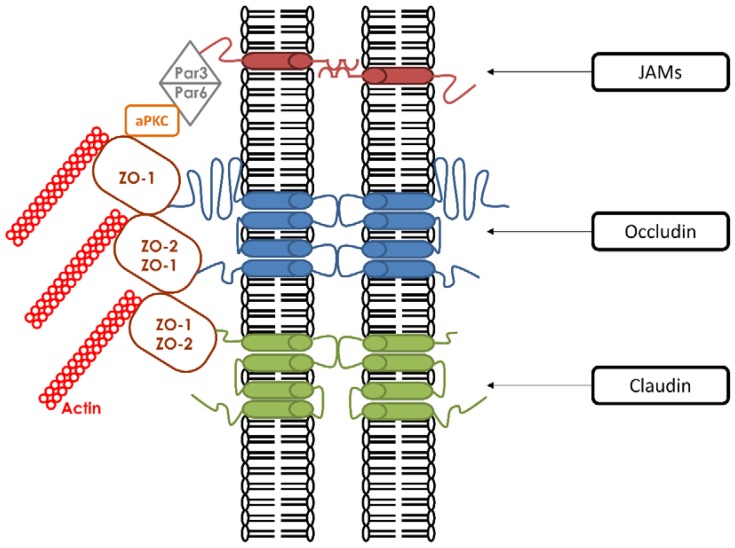
Molecular components of tight junctions. They are composed of three families of transmembrane proteins that include Occludins, Claudins, and Junctional Adhesion Molecule (JAMs). Every transmembrane protein is associated with cytoplasmic adaptor proteins such as the zonula occludens proteins ZO-1 and ZO-2. These interactions are mediated through their cytosolic tails. Other interactions occur between ZO proteins and additional cytoplasmic proteins (Par6, Par3, and aPKC). ZO proteins are also connected with the actin cytoskeleton.

TJ have two main roles. First, they demarcate the apical and basolateral domains of polarized cells by acting like a fence [22]. TJ prevent membrane proteins from diffusing freely between the two membrane compartments. This function is absolutely required in order for apical and basolateral domains to maintain their distinct lipid and protein compositions [23]. Second, they create a physiological and structural paracellular barrier that regulates the selective passage and exchange of molecules [24,25,26,27]. In many situations, various materials are selectively transported across cellular sheets, and this occurs either by direct transcellular transport or by paracellular flux through TJ. The selective passage of these components through TJ is mediated by aqueous pores whose structures have yet to be fully defined [28]. In addition to these functions, TJ are connected to signaling pathways that communicate with the cell cytoplasm and subcellular components [29]. To be able to perform all of these diverse functions, TJ must possess a complicated architecture based upon a multiprotein complex that is composed of more than 40 proteins that are classified either as transmembrane proteins or as cytoplasmic proteins bound to the actin cytoskeleton [30] (Figure 1).

### 1.1. Transmembrane Proteins

The three main transmembrane proteins are claudins [31], occludins [32], and Junctional Adhesion Molecules (JAMs) [33]. Both claudins and occludins contain four transmembrane regions (Tetraspans) with their amino-and carboxyl-terminal ends directed to the cytoplasm [34]. There are two isoforms of occludin generated by alternative splicing [35], whereas claudins comprise more than 24 members [36]. The extended C-terminus of claudins and occludins have been shown to be essential for interactions with the soluble cytoskeletal ZO proteins. These interactions mediate the association of the ZO proteins with the plasma membrane, which is an obligate step in the formation of TJ [37]. In vivo studies revealed that claudins are more necessary for the structural integrity of TJ than occludins. In occludin knock-out (KO) mice, TJ were morphologically intact [38] in comparison to claudin-KO mice, which showed the disappearance of TJ strands [39,40]. These results indicated that claudin is required for the formation of TJ strands, and suggested that occludin is rather required for TJ stability. The last category of transmembrane proteins are JAMs. They have a single transmembrane domain and their extracellular portion is folded into two immunoglobulin-like domains. Three isoforms are currently known: JAM-A [33], JAM-B, and JAM-C [41,42]. The three JAMs are co-distributed in epithelial cells with the ZO-1 protein. Evidence of a role for JAMs in TJ formation is supported by several studies. Overexpression of JAM proteins enhances the recruitment of TJ components and leads to the increased accumulation of ZO-1 and occludin [43].

### 1.2. Cytoplasmic Proteins

The “cytoplasmic plaque of TJ” serves as a link between the transmembrane TJ proteins and the actin cytoskeleton [44]. Most of the cytoplasmic proteins can attach to the TJ plaque via PDZ-domains. A PDZ domain is a common structural domain that interacts with stereotypical sequences embedded within the C-terminal regions of transmembrane proteins. PDZ domain-containing proteins can interact with other PDZ domain-containing proteins and through these multiplexed associations can anchor TJ membrane proteins to the cytoskeleton. PDZ domains are implicated in a variety of signaling mechanisms [45]. The two most important PDZ-containing proteins identified at the TJ plaque are the zonula-occludens-(ZO)1 proteins, which belong to the membrane-associated guanylate kinase family (MAGUK), and the partitioning defective proteins (Par), members of the Par3/aPKC/Par6 polarity complex.

The MAGUK family includes three structurally related proteins: ZO-1, ZO-2, and ZO-3. ZO-1 was the first to be associated with TJ [21,46]. They all share a similar structural organization, with an N-terminal region containing three PDZ domains. In vitro as well as in vivo analyses showed that the first PDZ domain (PDZ1) of the three ZO proteins has binding affinities for the C-terminal domains of claudins [37]. This PDZ-dependent interaction with ZO proteins promotes the proper targeting of claudins to the TJ. Furthermore, ZO-1/ZO-2 knock-down (KD) cells show disruptions in claudin localization associated with barrier dysfunction [47,48,49]. The second PDZ domain (PDZ2) is responsible for dimerization with other ZO proteins [50]. The third PDZ domain (PDZ3) is associated with the interaction with JAM-A [43]. Surprisingly, these three PDZ domains are not sufficient for the recruitment of ZO proteins to TJ [51]. In addition to these three PDZ domains, ZO proteins also have other regions that are required for their recruitment to TJ. These include SH3 and GUK domains, which can interact with afadin and occludin, respectively [52].

Given the fact that occludin-deficient cells are able to form normal TJ, with the appropriate distribution of ZO-1 [53], alternative interactions must necessarily be involved. One possibility would be the interaction of ZO proteins with α-catenin, a cytoplasmic actin-binding protein that associates with the β-catenin/E-cadherin complex at AJ [54,55]. However, α-catenin-deficient cells are able to recruit ZO-1 to the plasma membrane, indicating that this interaction is not critical. The final hypothesis focuses on the nectin/afadin complex and the specific interaction of the proline-rich regions of afadin with the SH3 domain of ZO proteins. Interaction between afadin and ZO-1 during the formation of cell-cell junctions in MDCK cells has been reported [56]. This interaction is principally observed before TJ are formed. During and after the formation of TJ, ZO proteins appear to be dissociated from afadin, and afadin becomes associated with nectin at AJ [57,58,59,60]. The association between TJ components and the AJ complex (α-catenin/β-catenin/E-cadherin and nectin/afadin) thus appears to catalyze the deposition of TJ proteins to the cell surface in the early steps of TJ formation. In summary, ZO proteins are essential for TJ formation, as well as for the linking of TJ membrane proteins to the actin cytoskeleton [51,61].

Besides the MAGUK family, members of the Par family play key roles in TJ assembly. Observations on the asymmetric divisions occurring in the *C. elegans* zygote led to the discovery of six Par proteins by Kemphues et al. in 1988, which are essential for the partitioning of early determinants and the development of embryonic polarity [62]. Only Par3 (Also known as Bazooka) and Par6 were found to be colocalized in *C. elegans* embryos. They both contain a PDZ domain and are able to bind to each other [63]. Par6 contains both N-terminal and C-terminal regions and three conserved domains for their interactions with other members of this complex. Its first domain PB1 (Phox/Bem 1) is located at the N-terminal region and is essential for the interaction of Par6 with atypical protein kinase C (aPKC). The second is Cdc42/Rac interaction binding (CRIB) and can be directly modulated by the cell division control protein 42 (Cdc42). The third one is a PDZ domain located at the C-terminal region. Accumulating evidence showed that Par3 and Par6 function together with aPKC [64]*.* The PB1 domain of Par6 binds the PB1 domain of aPKC to form a heterodimer. Par3 also contains N-terminal and C-terminal regions separated by three central PDZ domains. This tripartite Par3/aPKC/Par6 is known as the “Par complex” and is conserved from worms to vertebrates [65,66]. This interaction is a membrane targeting signal. In the Par complex, Par3 associates with the Par6/aPKC heterodimer by a PDZ-PDZ domain interaction at the onset of epithelial polarization [67]. Several molecules, such as nectin and JAMs, can bind the PDZ1 domain of Par3.

## 2. AMPK Is a Key Regulator of Energy Balance

**Figure 2:**
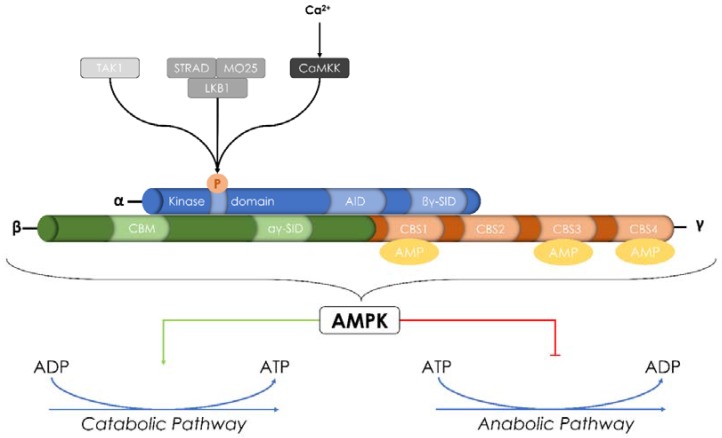
Structure and activation of AMP-activated protein kinase (AMPK). AMPK is a heterotrimeric α-β-γ serine/threonine kinase. It is made up of a catalytic α-subunit complexed with regulatory β-and γ-subunits. It can be activated through the phosphorylation of Thr-172 by two main upstream kinases: Ca^2+^-Calmodulin Kinase Kinase (CaMKK) and Liver Kinase B1 (LKB1). Transforming growth factor-β-activated kinase (TAK1) was also described as a new AMPK regulatory kinase. In addition to its activation by upstream kinases, AMPK can also be allosterically activated by AMP. Once activated, AMPK responds to changes in the level of Adenosine triphosphate (ATP) by switching off either anabolic and biosynthetic pathways consuming ATP or switching on catabolic pathways that produce ATP.

Each accomplish energy-requiring tasks through the hydrolysis of ATP into ADP, which serves as their immediate source of energy [68]. Maintaining an adequate supply of energy is an essential requirement for survival, which means that ATP levels must be kept at a sufficient concentration. The main sensor of cellular energy status is the AMP-activated protein kinase (AMPK). When ATP levels fall, its main function is to switch off anabolic and biosynthetic pathways that consume ATP and to switch on catabolic pathways that produce ATP [69] (Figure 2). When the overall energy levels in cells decrease due to increased demands or decreased availability of substrates, AMPK gets activated through a combination of phosphorylation by upstream kinases and/or direct activation by AMP and ADP [70,71].

### 2.1. AMPK: Structure and Regulation

AMPK is a heterotrimeric serine/threonine kinase. It is made up of a catalytic α-subunit complexed with regulatory β-and γ-subunits [72]. There are 12 unique heterotrimeric combinations of AMPK. In mammals, the α-subunit is encoded by two isoforms, and the β-and γ-subunits are encoded by two and three isoforms, respectively (α1, α2, β1, β2, γ1, γ2, and γ3). All these isoforms have differential tissue-specific expression and activity [73,74,75].

The α-subunit possesses an *N*-terminal kinase domain that mediates its catalytic activity and a *C*-terminal subunit-interacting domain that plays a role in the interaction with β-and γ-subunits (βγ-subunit interacting domain-βγ-SID) [76]. The α1-subunit is expressed in many organs (kidney, heart, brain, spleen, liver, lung, and skeletal muscle), unlike α2-subunit, which is essentially expressed primarily in skeletal muscle [77]. In addition to their different tissue/organ expression, α1- and α2-subunits are differentially expressed within the cell. Indeed, α1 is predominantly expressed in the cytosol, whereas α2 is localized to the nucleus in periods of high energy demand [78]. The β-subunit contains a central glycogen-binding domain CBM (carbohydrate-binding module) that permits the interaction of AMPK with glycogen particles and a *C*-terminal region essential for the assembly of the α β γ complex [79]. The γ-subunit contains four cystathionine-β-synthase (CBS) tandem sequence repeats that fold to form two “Bateman domains” and can bind AMP or ATP to regulate the AMPK activation [80] (Figure 2).

Phosphorylation of Thr-172 in the α-subunit catalytic loop is the main pathway that produces the activation of AMPK. Nevertheless, there are three major mechanisms responsible for the AMPK activation: (i) upstream kinases [71]; (ii) the increase of [AMP] and/or [ADP] [81,82]; and (iii) direct binding to the γ-subunit of AMP for the allosteric activation of AMPK [83]. Beside its activation by allosteric AMP binding and upstream kinases, AMPK has been reported to have an autophosphorylation ability at β-subunit Thr-148 [84,85].

### 2.2. AMPK: Upstream Kinase and Substrates

Two major upstream AMPK-regulatory kinases have been discovered that are both serine/threonine kinases. The first one is the liver-kinase-B1 (LKB1) and the second one is the Ca^2+^/calmodulin-dependent kinase kinase (CaMKKβ). LKB1 and CaMKK can activate AMPK in response to energy stress as signaled by elevated AMP levels or to increases of intracellular [Ca^2+^] levels in an AMP-independent manner, respectively. Other studies have also demonstrated that transforming growth factor-β-activated kinase (TAK1) may represent a third AMPK kinase.

LKB1 phosphorylates and activates AMPK in vitro following increased cellular [AMP] levels [86]. LKB1 activity requires the binding of the scaffolding-related adaptor mouse protein 25 (MO25) and the pseudokinase STe-20 Related ADaptor (STRAD) via the formation of the holoenzyme complex [86]. In cells lacking the expression of LKB1, the activation of AMPK in response to the increase of the AMP/ATP ratio is abolished, suggesting that LKB1 is required for the AMPK phosphorylation when [AMP] increases in the cell [86]. Other studies demonstrated that, in certain circumstances, AMPK can be activated, even in the absence of LKB1 [87,88]. Hence, CaMKK emerged as another main AMPK kinase [89]. In contrast to LKB1, the AMPK phosphorylation by CaMKK does not require a disturbance of the ATP/AMP ratio, but rather an increase in intracellular Ca^2+^ [90]. The addition of the Ca^2+^ ionophore A23187 activates AMPK-via the phosphorylation of Thr-172-approximately 10-fold more in cells expressing a kinase-inactive mutant of LKB1 compared to wild-type cells. Conversely, the AMPK activation by Ca^2+^ ionophore A23187 was abolished by the CaMKK inhibitor STO-609 [91]. Other studies confirmed this result and observed that the overexpression of CaMKK increases AMPK activity, whereas the inhibition of CaMKK reduces AMPK activity [92]. These results suggest a physiological role of LKB1 and CaMKK as AMPK regulatory kinases in mammalian cells [92].

AMPK is a modulator of several pathways [93,94,95]. For example, AMPK negatively regulates two enzymes involved in lipid synthesis: HMGCR (3-hydroxy-3-methylglutaryl coenzyme A reductase) [96] and ACC (acetyl-CoA carboxylase) [97]. AMPK also exerts a potent effect on glucose metabolism. Glucose uptake is facilitated through the translocation of glucose transporter 4 (GLUT4) to the cell membrane and also through the regulation of *GLUT4* gene expression in response to AMPK activation [98]. AMPK also regulates glycogen metabolism. AMPK activation phosphorylates and decreases the activation of glycogen synthase (GS), thus reducing glycogen synthesis [99]. Thus, AMPK activation reduces the production of stored forms of metabolic energy and diminishes the activity of energy utilizing pathways, while it increases the capacity of cells to import energetic precursors and to produce ATP. Kishton et al. showed that AMPK actively restrained aerobic glycolysis in cells through the inhibition of mTORC1, while promoting oxidative metabolism and mitochondrial Complex I activity producing ATP [100]. The inhibition of AMPK-related kinase 5 (ARK5), an upstream regulator of AMPK, leads to a collapse of cellular ATP levels. Proteomics highlighted the down-regulation of multiple subunits of complexes I, III, and IV of the mitochondrial respiratory chain following the depletion of ARK5 [101]. These studies suggest a role of AMPK in the production of ATP by the mitochondrial respiratory chain. AMPK facilitates the assembly of TJ.

## 3. AMPK and ZO-1

Zhang et al. [17] demonstrated in 2006 that AMPK could regulate the assembly of epithelial TJ in the MDCK cell line. The authors used a Ca^2+^ switch-based model described in 1978 [102] to decipher the role of AMPK in TJ assembly. Cell-cell adhesion, as well as TJ integrity in polarized epithelial cells, is rapidly lost when the Ca^2+^ is removed from the extracellular medium. On the other hand, the re-addition of Ca^2+^ into the culture medium induces the rapid assembly of cell-cell contacts and subsequent TJ formation. Depletion of Ca^2+^ from the medium causes ZO-1 to translocate from the cell periphery to the cytoplasm. Upon the re-addition of Ca^2+^, ZO-1 moves back to the TJ. With this model, the authors showed that AMPK is phosphorylated during the Ca^2+^-induced TJ assembly, while the total amount of AMPK remains unchanged. They also examined the AMPK activity by measuring the phosphorylated form of ACC, one of the principle AMPK substrates. They found an eight-fold increase in pACC following a Ca^2+^-switch. It is important to note that such AMPK phosphorylation and activation was not attributable to changes in cellular [ATP] levels during a Ca^2+^-switch. To evaluate the potential effect of AMPK in TJ formation, the authors monitored the time course of ZO-1 relocation to cell-cell junctions with or without the chemical AMPK activator AICAR (which acts an AMP mimic) added at the time of the Ca^2+^ switch. The amount of ZO-1 relocated to TJ in the presence of AICAR during the Ca^2+^ switch was higher in comparison to a classic Ca^2+^ switch. Furthermore, the addition of AICAR in Ca^2+^-depleted medium was sufficient to activate AMPK and to accelerate ZO-1 relocation to TJ [17]. They also measured the paracellular flux of 70-kDa dextran in MDCK monolayers during the Ca^2+^-switch in the presence or absence of AICAR. The presence of AICAR led to a slight but statistically significant decrease in the dextran flux rate across the monolayers. Similarly to Zhang et al., Peng et al. also measured the paracellular flux by measuring inulin in intestinal epithelial cells with or without AICAR. Incubation with AICAR led to a significant decrease in the flux rate across the cell monolayers. These effects were also abolished by the AMPK inhibitor, Compound C. These studies indicated that the backleak effect is decreased by the activation of AMPK, independently of [ADP/ATP] changes. In MDCK cells expressing dominant negative AMPK, the early initiation of TJ assembly was compromised. Still, normal-appearing TJ could eventually form in AMPK-deficient cells over time, suggesting that AMPK activation supports the initiation of TJ formation, but other factors, including extracellular Ca^2+^, are required for the long-term stabilization of TJ. Shortly after the publication of the Zhang et al. study, Zheng et al. confirmed these findings and in addition showed that the activation of AMPK in response to the initiation of junction formation requires LKB1. They also generated MDCK cell lines expressing a kinase-dead mutant form of AMPKα1 and monitored the effect of Ca^2+^-switch on TER, a measurement for the paracellular barrier function and integrity of TJ [103]. Expression of kinase-dead AMPK significantly decreased the peak level of TER, meaning that the formation of functional TJ is suppressed in the absence of AMPK [104].

## 4. AMPK and Afadin-Nectin System

In the above-detailed model, the nectin-afadin system is required for the deposition of junction components induced by AMPK activation. The involvement of the nectin-afadin complex in cell adhesion has been described in AJ formation [105]. Still, afadin KD cells are not able to induce the relocalization of ZO-1 and occludin at TJ sites following the addition of AICAR in low Ca^2+^ medium. AMPK therefore appears to be connected to afadin in the Ca^2+^-independent TJ formation. Furthermore, immunoprecipitation between afadin and AMPK revealed that afadin is a direct substrate of AMPK. The addition of Compound-C inhibited the phosphorylation of afadin, whereas the afadin signal was increased without the AMPK inhibitor [106]. Since afadin directly binds to ZO-1 [56], the authors investigated whether AMPK activation increases the interaction between afadin and ZO-1, thereby facilitating the assembly of TJ. Immunoprecipitation, with or without AICAR during a Ca^2+^ switch, revealed an enhanced interaction between these two proteins after Ca^2+^-switch and even more in the case of AICAR exposure. These results suggested that AMPK activation might facilitate TJ assembly by phosphorylating afadin and inducing its association with ZO-1.

## 5. AMPK Effectors

Recent research investigated the potential of AMPK effectors to preserve the epithelial architecture. The first study focused on the multimodular polarity scaffold protein GIV (G-alpha interacting vesicle associated protein) [107]. This protein has been demonstrated to regulate cell polarity and morphogenesis [108], as well as cell-cell junction formation through its ability to bind Par3 [109] and the Cadherin-catenin complex [110]. A role for AMPK-mediated phosphorylation of GIV at serine 245 when [ATP] levels decreased was suggested. The phosphorylation of GIV at ps 245 triggered its localization to TJ by increasing its ability to bind TJ-associated microtubules and AJ-localized protein complexes. The addition of Compound-C inhibited AMPK-mediated phosphorylation of GIV and induced the destabilization of TJ and the reduction of TER. On the other hand, metformin (an AMPK activator) and AICAR triggered GIV phosphorylation and stabilized TJ, with subsequent enhanced TER [107].

In 2005, butyrate emerged as a new candidate to promote enhanced intestinal barrier function as reflected by increases in TER in vitro [111]. A few years later, Peng and co-workers explored whether the effect of butyrate on the intestinal epithelial barrier is related to AMPK. Using a model of Ca^2+^ switch, with or without the addition of butyrate, they demonstrated that the amount of pAMPK, as well as pACC, increased after the treatment with butyrate in a time-dependent manner. The addition of AICAR in the culture medium increased TER, whereas Compound-C abolished this effect. Butyrate also promoted a faster relocalization of ZO-1 and occludin at the cell periphery and tightened the intestinal barrier [112]. These results further support a role for AMPK activation in TJ formation. Additional studies using sodium butyrate further support the AMPK activation by CaMKK due to the increasing of the intracellular concentration of Ca^2+^. Furthermore, the AMPK activation also increases the phosphorylation of PKCβ, a key player in TJ regulation [65,113]. This study underscores the putative interplay between the AMPK and PKC family in the formation of TJ [114].

Another model involving porcine intestinal epithelial cells investigated the effect of L-glutamine (Gln) in the preservation of TJ. Gln was described as a critically important nutrient for the maintenance of intestinal mucosal barrier integrity in humans and animals [115]. Indeed, the depletion of Gln results in a decreased abundance of TJ-associated proteins and increased intestinal paracellular permeability, whereas the addition of Gln resulted in increased TER, enhanced TJ-protein abundance, and the localization of TJ proteins to the plasma membrane [116]. TJ proteins, such as claudin-1, claudin-4, and ZO-1, are localized more abundantly at TJ sites in the presence of Gln. In addition, the abundance of pAMPK was further enhanced by the addition of Gln. The beneficial effect of Gln, as well as the phosphorylation of AMPK, was abrogated in a low Ca^2+^ medium and with the use of STO-609 (CaMKK inhibitor). Gln increased the intracellular [ATP] levels, but these were not affected by STO-609, meaning that AMPK acts as a TJ regulator via the CaMKK pathway in a model of Ca^2+^-induced TJ formation [117]. Our recent work further supports a similar role for CaMKK in the activation of AMPK during a Ca^2+^ switch. This study found that the pharmacological inhibition of CaMKK or the direct inhibition of AMPK by Compound-C hampered AMPK phosphorylation and ZO-1 relocation to the TJ during a Ca^2+^ switch in MDCK cells, whereas the inactivation of LKB1 by shRNA did not significantly influence these processes [118].

Park et al. focused their research on the beneficial effect of Theaflavins (TFs), a polyphenol pigment in black tea, known to have anti-hyperglycemic, antioxidant, and anti-inflammatory effects. They previously found that TFs induced AMPK activation. By measuring the fluorescein transport across epithelial cells, they observed a decrease in its transport by pre-treatment with TFs, thereby revealing a reduction of paracellular permeability. Moreover, the TJ-related proteins claudin-1, occludin, and ZO-1 were significantly increased at TJ. Furthermore, Compound-C restored the fluorescein transport and inhibited the action of TFs. The authors highlighted the AMPK-mediated expression of claudin-1, occludin, and ZO-1 at TJ in intestinal cells by TFs [119]. Another natural agent, i.e., Forskolin, has been demonstrated to have beneficial effects on the AMPK-mediated TJ formation. This compound increased the phosphorylation and activation of AMPK with a comparable effect to the addition of 2 Deoxy-D-Glucose (2-DG) in a placenta epithelial cell culture. Forskolin treatment markedly enhanced the assembly of TJ strands, with higher ZO-1 relocation at TJ, while only weak ZO-1 staining was observed in control cells. The authors also used dominant negative AMPK transfected cells or Compound-C to inhibit AMPK activation and examined whether or not Forskolin-induced TJ assembly is mediated by AMPK activation. In both cases, the Forskolin effect was abrogated, resulting in lower ZO-1 relocation at TJ [120]. Thus, the activation of AMPK by Forskolin may enhance TJ formation.

AMPKα-null *Drosophila* die before reaching adulthood, while the transgenic expression of wild-type AMPK in AMPKα-null mutants allowed them to successfully develop into adults. A detailed examination of the embryonic epithelial structure of AMPKα-null mutants revealed a major disorganization of apico-basal polarity. They also assessed whether AMPK is necessary for cell polarity in mammalian cells. 2-DG treatment of unpolarized epithelial cells, such as the LS174T line, induced major changes in cell shape with the formation of a polarized actin cytoskeleton and a brush-border-like structure. Interestingly, this actin polarization was suppressed by the AMPK-specific inhibitor Compound C [121]. Furthermore, the authors have shown that AMPKα mutation in *Drosophila* embryos leads to the abnormal distribution of epithelial polarity markers. The consequent loss of polarity along with over-proliferative aberration could promote cancers [122]. In addition, AMPK has been shown to suppress tumorigenesis and the Warburg effect [123]. Therefore, one may speculate that AMPK-mediated TJ strengthening may help inhibit adenocarcinoma and tumorigenesis. Additional studies involving AMPK KO mice showed higher intestinal permeability when compared with WT mice, as indicated by decreased TER and increased paracellular FITC-dextran permeability, indicating a leaking gut. To investigate the integrity of TJ, ZO-1 immunofluorescence staining was analyzed and ZO-1 labeled at the tip of villi was impaired in AMPK KO mice [124].

Another role of AMPK in TJ formation and maintenance could be hypothetically linked to dietary methionine restriction (MR). MR has been found to modify the protein composition of TJ complexes in epithelial cells [125]. In addition, the stimulation of *S-*adenosyl-l-methionine, a key intermediate of methionine metabolism, led to the consumption of both Met and ATP [126] and AMPK activation [127]. These two studies may suggest a new role of AMPK in TJ maintenance in the case of MR. Along with other nutritional regimens modulating AMPK, Zinc has the potential to function as a TJ modifier and selective enhancer of epithelial barrier function [128] by regulating claudin-3 and occludin [129]. Since the rapid activation of AMPK was observed after exposure of neurons to Zinc, one may speculate an interplay between zinc-induced TJ formation and the AMPK pathway. Interestingly, Zinc-induced AMPK activation was mediated by LKB1 in the absence of changes in intracellular AMP levels or CaMKKβ activation [130].

## 6. AMPK and Co-Culture Models

The involvement of AMPK in TJ formation has also been demonstrated in several models of direct cell-cell co-culture. Tang et al. investigated the role of lymphocytes in the modulation of the epithelial barrier since lymphocytes are recruited by epithelial cells during infection. To mimic an infection state, they used a direct co-culture of MDCK cells with lymphocytes and a Ca^2+^ switch model to measure the TJ formation. The time course of ZO-1 relocation after Ca^2+^ switch was accelerated in the co-culture compared to MDCK alone. Furthermore, lymphocytes drastically increased AMPK phosphorylation in comparison to MDCK alone after a Ca^2+^-switch. To link the increased AMPK phosphorylation and TJ formation, the authors used Compound-C and MDCK expressing an shRNA directed against AMPKα1. In both cases, the beneficial effect of lymphocytes was abolished and a slower TJ assembly was observed, thereby confirming the requirement for AMPK in the TJ formation [131]. Similar experiments were performed in a co-culture model of mesenchymal stromal cells (MSC) and MDCK cells. Bone marrow-derived MSC can modulate epithelial TJ at the time of their Ca^2+^-induced assembly. The relocation of ZO-1 to MDCK cell-cell contacts was indeed significantly accelerated in the presence of MSC compared to a MDCK cell culture alone. Furthermore, AMPK activation and activity were also enhanced in the co-culture model. The addition of Compound-C or STO-609 abolished this AMPK activation and ZO-1 relocation. On the other hand, the co-culture of MSC with MDCK expressing an shRNA directed against LKB1 did not suppress the AMPK activity and ZO-1 relocation. This work further supports a role for CaMKK in the activation of AMPK and ZO-1 protein relocation at TJ during a Ca^2+^-switch, independently of LKB1 activity [118].

Patkee et al. worked on metformin and its role in airway epithelial TJ in a model of co-culture with *P. aeruginosa* (a respiratory pathogen) to mimic an infection and TJ disruption with higher glucose permeability. The addition of *P. aeruginosa* into the culture of airway epithelial cells produced a significant decrease in TER. Metformin treatment attenuated the fall in TER produced by *P. aeruginosa*. AICAR pre-treatment also attenuated the *P. aeuringosa*-induced reduction of TER. On the other hand, this increasing TER was prevented by pre-treatment with the AMPK inhibitor Compound-C. To explain this increased TER phenomenon, the authors investigated the effect of *P. aeruginosa* and metformin on the abundance of TJ proteins. They found a decline in claudin-1 and occludin abundance in the co-culture with *P. aeruginosa.* The addition of metformin enhanced the expression of these two TJ proteins. These data indicate a potential AMPK-dependence that may be responsible for metformin’s ability to increase the airway epithelial barrier function [132].

## 7. Conclusions

**Figure 3:**
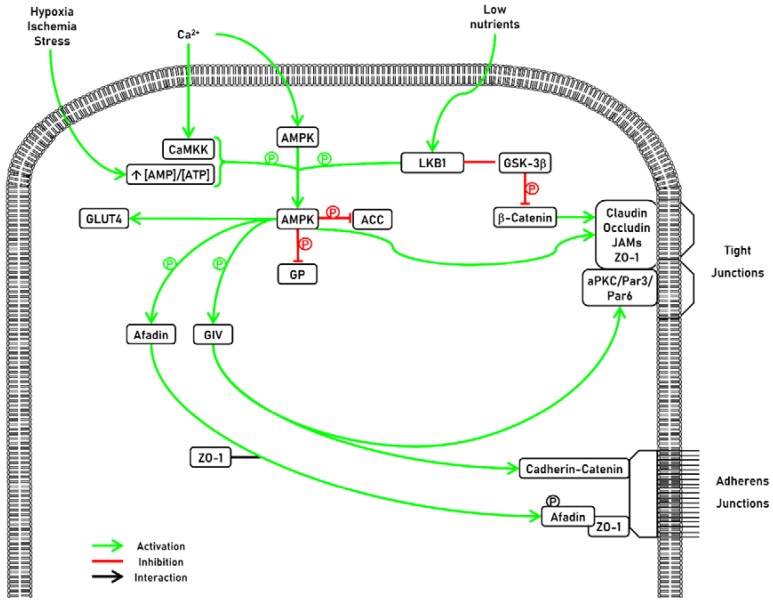
Representative schema of AMPK activators and substrates.

TJ are key constituents of polarized epithelial cells. It is well established that the presence of TJ is indispensable for tissue compartmentalization and cellular homeostasis. Their disruption represents one of the earliest markers of epithelial injury and diseases. Accumulating evidence demonstrates that AMPK is a key factor in the formation of TJ via several signaling pathways (Figure 3). Further investigations concerning the impact of AMPK on epithelial maintenance in baseline conditions and in diseased conditions may lead to innovative therapies.

AMPK is involved in tight junctions (TJ) formation. AMPK can be activated by two mains upstream kinases: Ca^2+^-Calmodulin Kinase Kinase (CaMKK) and Liver Kinase B1 (LKB1). Once activated, AMPK can have several effects. First, AMPK can modulate lipid metabolism by targeting the fatty acid synthesis pathway by the phosphorylation and inhibition of Acetyl CoA Carboxylase (ACC). Second, AMPK also exerts a potent effect on glucose metabolism trough the translocation of glucose transporter 4 (GLUT4) to the cell membrane. Third, AMPK also regulates glycogen metabolism. AMPK activation phosphorylates and decreases the activation of glycogen synthase (GS), thus reducing glycogen synthesis. AMPK is involved in TJ formation. Activated AMPK phosphorylates afadin and induces its association with ZO-1. AMPK also phosphorylates G-alpha interacting vesicle associated protein (GIV), which regulates cell polarity and morphogenesis, as well as cell-cell junction formation through its ability to bind Par3 and the Cadherin-catenin complex.
